# Modeling the SDF-1/CXCR4 protein using advanced artificial intelligence and antagonist screening for Japanese anchovy

**DOI:** 10.3389/fphys.2024.1349119

**Published:** 2024-02-02

**Authors:** Issei Yahiro, Kyle Dominic Eguid Barnuevo, Oga Sato, Sipra Mohapatra, Atsushi Toyoda, Takehiko Itoh, Kaoru Ohno, Michiya Matsuyama, Tapas Chakraborty, Kohei Ohta

**Affiliations:** ^1^ Laboratory of Marine Biology, Faculty of Agriculture, Kyushu University, Fukuoka, Japan; ^2^ Aqua-Bioresource Innovation Center, Kyushu University, Saga, Japan; ^3^ Advanced Genomics Center, National Institute of Genetics, Shizuoka, Japan; ^4^ School and Graduate School of Bioscience and Biotechnology, Tokyo Institute of Technology, Kanagawa, Japan; ^5^ National Institute for Basic Biology (NIBB), Aichi, Japan

**Keywords:** SDF-1/CXCR4, Colab Fold, CB Dock, Alpha Fold, PGC, Japanese anchovy

## Abstract

SDF-1/CXCR4 chemokine signaling are indispensable for cell migration, especially the Primordial Germ Cell (PGC) migration towards the gonadal ridge during early development. We earlier found that this signaling is largely conserved in the Japanese anchovy (*Engraulis japonicus*, EJ), and a mere treatment of CXCR4 antagonist, AMD3100, leads to germ cell depletion and thereafter gonad sterilization. However, the effect of AMD3100 was limited. So, in this research, we scouted for CXCR4 antagonist with higher potency by employing advanced artificial intelligence deep learning-based computer simulations. Three potential candidates, AMD3465, WZ811, and LY2510924, were selected and *in vivo* validation was conducted using Japanese anchovy embryos. We found that seven transmembrane motif of EJ CXCR4a and EJ CXCR4b were extremely similar with human homolog while the CXCR4 chemokine receptor N terminal (PF12109, essential for SDF-1 binding) was missing in EJ CXCR4b. 3D protein analysis and cavity search predicted the cavity in EJ CXCR4a to be five times larger (6,307 Å³) than that in EJ CXCR4b (1,241 Å³). Docking analysis demonstrated lower binding energy of AMD3100 and AMD3465 to EJ CXCR4a (Vina score −9.6) and EJ CXCR4b (Vina score −8.8), respectively. Furthermore, we observed significant PGC mismigration in microinjected AMD3465 treated groups at 10, 100 and 1 × 10^5^ nM concentration in 48 h post fertilized embryos. The other three antagonists showed various degrees of PGC dispersion, but no significant effect compared to their solvent control at tested concentrations was observed. Cumulatively, our results suggests that AMD3645 might be a better candidate for abnormal PGC migration in Japanese anchovy and warrants further investigation.

## 1 Introduction

Aquaculture plays an important role in global food production by efficiently producing high-quality animal protein (Sargent and Tacon, 1999; Subasinghe et al., 2009; Trygve et al., 2012). In the quest for more efficient development of fish varieties, and better production, the science of fish reproduction has made remarkable progress in recent decades (Edward and George, 1982; Francesc, 2001; de Siqueira-Silva et al., 2018; Goto and Saito, 2019). The inhibition of natural reproduction via germ cell reduction offers additional benefits for fish, including improved growth, meat quality, and disease resistance (Ali and Rao, 1989; Zohar, 1989). Moreover, gonadal sterilization is expected to be an effective method for preventing genetic contamination in the natural environment (Nagasawa et al., 2019), eliminating invasive species (Roger et al., 2003), and protecting the intellectual property rights of highly bred varieties (Xu et al., 2023). Despite their importance, successful gonadal sterilization has been achieved in only handful of species, and most of them through targeting the primordial germ cell (PGC), the putative precursor of germ cells (Aleksandar et al., 2016), migration and development (Wong and Zohar, 2015). Moreover, mass-scale and cost-effective gonad sterilization technologies are very limited and utmost necessary for aquaculture adaptability.

In fish, PGC formation occurs at multiple locations during the early stages (starts around 4 cells stage) of development (Raz, 2004), migrate to the gonadal ridge by somite formation stage and initiates gonadal development to transmit genetic information to the next-generation (Raz, 2003). SDF-1 (Stromal cell-derived factor 1, or CXCL12)/CXCR4 (C-X-C chemokine receptor type 4, or CD184) is a type of chemokine signaling that is involved in many types of cell migration, including PGC migration. Inhibiting SDF-1/CXCR4 signaling has been reported to have significant effects on PGC migration, gonad development and sexuality, and gonad sterilization of medaka and other fish (Holger et al., 2003; Kurokawa et al., 2007; Herpin et al., 2008; Wong and Collodi, 2013; Chakraborty et al., 2019). Similarly, using Japanese anchovy, we found that inhibition of either SDF-1 or CXCR4, disrupts germ cell settlement in the gonad, and the later one even produces germ cell-less adult individuals (Yahiro et al., 2023; submitted elsewhere). However, the variability was very high that warrants better alternative.

Japanese anchovy (*Engraulis japonicus*, EJ) is an excellent upcoming marine model fish with several advantages like easy breeding and rearing, small size, fast maturity, and shorter generation time (Sakaguchi et al., 2019). Until recently, *in silico* analysis of protein structures in non-mammalian models such as Japanese anchovy was challenging. However, recent advanced algorithmic tools like Alpha Fold2 (Senior et al., 2020; Jumper et al., 2021), Colab Fold (Mirdita et al., 2022) and CB Dock2 (Liu et al., 2022; Yang et al., 2022) have been developed. AlphaFold2 and ColabFold employs artificial intelligence and deep learning to accurately predict protein structure, and CB-Dock2 uses advanced computational algorithms to predict cavity and antagonist binding, both with remarkable accuracy and ease. These could accelerate not only the research of human or drug discovery, but also the research using non-mammalian model organisms. So, in this study, we focused on screening various readily available CXCR4 antagonists used in human medicine and cancer research for their better compatibility in the PGC migration physiology of Japanese anchovy. Four comparably low-cost, readily available, and proven CXCR4 antagonists, i.e., AMD3100 (Sigrid et al., 2002), AMD3465 (Hatse et al., 2005), WZ811 (Li et al., 2016), and LY2510924 (Galsky et al., 2014), were used in this study. We first predicted 3D structures for SDF-1/CXCR4 protein of Japanese anchovy (EJ SDF-1/EJ CXCR4) using Colab Fold, obtained a reference protein 3D structure model, and performed a cavity search using CB Dock. In addition, we compared their suitability of EJ CXCR4 by performing auto blind docking using CB Dock and compared the Vina Scores (Trott and Olson, 2010), which indicates the binding free energy (a composite factor including Gaussian force, hydrophobicity, and hydrogen bonding). Further, we aimed to clarify the effects of each antagonist on PGC migration and identify the most suitable antagonist for Japanese anchovy by using microinjection and immersion treatment, respectively, to ensure antagonist delivery into the eggs and to check their applicability in mass scale induction of PGC mismigration.

## 2 Materials and Methods

### 2.1 Sequence information, alignment, and motif analysis

Amino acid sequences of Japanese anchovy SDF-1a (WKC57597.1), SDF-1b (WKC57598.1), CXCR4a (WKF24609.1), CXCR4b (WKF24610.1), human (*Homo sapiens*) SDF-1 (NP_000600.1), CXCR4 (NP_003458.1), and zebrafish (*Danio rerio*) CXCR4a (NP_571957.2), CXCR4b (NP_571909.1), SDF-1a (NP_840092.1), SDF-1b (NP_001307343.1) were obtained from NCBI. Amino acid sequence alignment was performed in CLUSTAL W 2.1 with default setting (Slow/Accurate), and motif search was performed in MOTIF Search with default setting in the Pfam database.

### 2.2 Protein modeling

For protein 3D model, an online server, Colab Fold, based on Alpha Fold2 (Colab Fold v1.5.3: Alpha Fold2 using MMseqs2) was used for simulation, with default parameters. The resulting PDB files were edited and visualized in UCSF Chimera (Pettersen et al., 2004) and subsequently used for secondary structure analysis. Cavity searches using CB Dock (Liu et al., 2022; Yang et al., 2022) for CXCR4 were also performed, and the cavity size of the receptor site was shown. These results of CXCR4 were also used for antagonist binding analysis that used auto blind docking of CB Dock. AMD3100 (ab120718, abcam, USA), AMD3465 (ab120809, abcam, USA), WZ811 (S2912, Selleck Chemicals, USA), and LY2510924 (S8505, Selleck Chemicals, USA) were used in this study. The 3D Conformer models (SDF file) used for docking simulations were obtained from PubChem, and PubChem CIDs were AMD3100 (65015), AMD3465 (483559), and WZ811 (11565518). On the other hand, LY2510924, which has a large molecular size, could not be used in the simulation analysis due to the large number of rotatable parts. The results obtained for the ligands are shown with surface, while the receptors are shown in cartoon form and colored according to hydrophobicity. Each binding site (amino acid residues) are listed in Supplementary Figure S1. Human CXCR4 (PDB ID: 3OE9 (Wu et al., 2010), 4RWS (Qin et al., 2015), N- terminus binding, PDB ID: 2N55 (Ziarek et al., 2017)) and SDF-1 (PDBID: monomer 2KEE (Veldkamp et al., 2009), dimer 4UAI (Smith et al., 2014)) protein were retrieved from available literature.

### 2.3 Fish maintenance, egg collection, and microinjection

The Japanese anchovies used for experiment in this study, were breed inhouse, at least for two generations, from a wild stock caught in Kagoshima Bay, Japan. Approximately 300 adult fish were kept in a 4-ton circular FRP tank with 15x daily water exchange. The water temperature and photoperiod were respectively maintained at 20°C–23°C and 11L:13D. Natural breeding occurred between 4 and 6 h after dark and eggs were collected from the outlet of tank. Good quality fertilized eggs (floating and transparent) were transferred to filter sterilized sea water and used for antagonist tests. PGC visualization and microinjection of Japanese anchovy followed previously established protocol (Yahiro et al., 2023; submitted elsewhere). Briefly, the fertilized eggs were collected and placed on a 1.5% agar gel with grooves measuring 0.47–0.57 mm width, filled with seawater to prevent desiccation. Microinjection needles were prepared using GD-1 (Narishige, Japan) micro glass capillaries, pulled and grind to achieve a tip diameter of 1 µm (Ohga et al., 2023). Visualization of PGC was performed by injecting *egfp*-*nanos*3 3′UTR mRNA (*egfp* mRNA, 100 ng/μL). The injection solution was mixed with phenol red (0.05% of final volume, Sigma-Aldrich, USA), adjusted to the required concentration using DPBS(-) (Gibco, USA), and injected into the cytoplasm of 1∼8 cell stage of embryos. After injection, eggs were incubated at 20°C with daily water exchange. All fluorescence observations were made using a stereo microscope MZ10F (Leica, Germany) and photomicrographs were taken with a Flex Cam C1 camera (Leica, Germany).

### 2.4 Microinjection of antagonist

To ensure direct entry and reliable dosing of antagonists, *egfp* mRNA solutions containing antagonists were microinjected. Stock solutions of antagonist were prepared in DPBS(-) (Gibco, USA) or DMSO (Dimethyl sulfoxide, Nacalai tesque, Japan), as specified by manufacturers, and diluted to required concentration before use. The concentrations of the various antagonists were selected based on IC50/EC50 from previous studies: AMD3100 13 nM (Schols et al., 1997), AMD3465 10 nM (Hatse et al., 2005), WZ811 1.2 nM (Zhan et al., 2007), LY2510924 0.26 nM (Peng et al., 2015), and higher concentrations were tested to overcome interspecies differences, if any. The solutions used in microinjection contained *egfp* mRNA, 0.05% phenol red, and various antagonists: AMD3100 (1.3, 13, 130, 1.3 × 10^3^, 1.3 × 10^4^, 1.25 × 10^5^, 6.0 × 10^5^ nM), AMD3465 (10, 100, 1.0 × 10^3^, 1.0 × 10^4^, 1.0 × 10^5^ nM), WZ811 (1, 10, 100, 1.0 × 10^3^nM) LY2510924 (1, 10, 100, 1.0 × 10^3^, 1.0 × 10^4^, 1.0 × 10^5^ nM), with final dilution adjustment using DPBS(-). Microinjected eggs were placed in sterile seawater (0.22 μm filter sterile) and incubated at 20°C until 48 h after fertilization (hpf). Solvent-control injected, and non-injected eggs were similarly incubated.

### 2.5 Immersion of antagonist

Immersion treatment was performed using AMD3465, WZ811, and LY2510924. Higher concentrations were used than in the microinjection method to compensate any loss during penetration. 1, 10, and 50 μM treatment water was prepared just before use, by adding required amount of antagonist in filter sterilized sea water. Seventeen fertilized *egfp* mRNA microinjected eggs with were placed in 5 mL of treatment water with regular water change at 24 h interval. Hatched larvae were anesthetized and observed under microscope. PGCs visualizations were performed as mentioned above. Solvent-control samples were similarly maintained and observed.

### 2.6 Distance between the farthest PGCs (DFP) analysis

To easily quantify the effect of antagonists on PGC migration, ImageJ Fiji were used (Schindelin et al., 2012). Distance between the farthest PGCs (the most distant PGCs in the body) in each of 6 randomly sampled individuals/experimental group were measured. The distances in pixels were converted to mm units, and used for analysis.

### 2.7 Figures and data analysis

Each *in vivo* experiment was conducted for 3 times. All data were graphed using Prism 9 (GraphPad, USA) and significance tests were performed using one-way ANOVA and multiple *post hoc* tests (Tukey test). Error bars in all figures show SEM.

## 3 Result

### 3.1 Analysis of EJ SDF-1/CXCR4 amino acid sequence and motif

**FIGURE 1:**
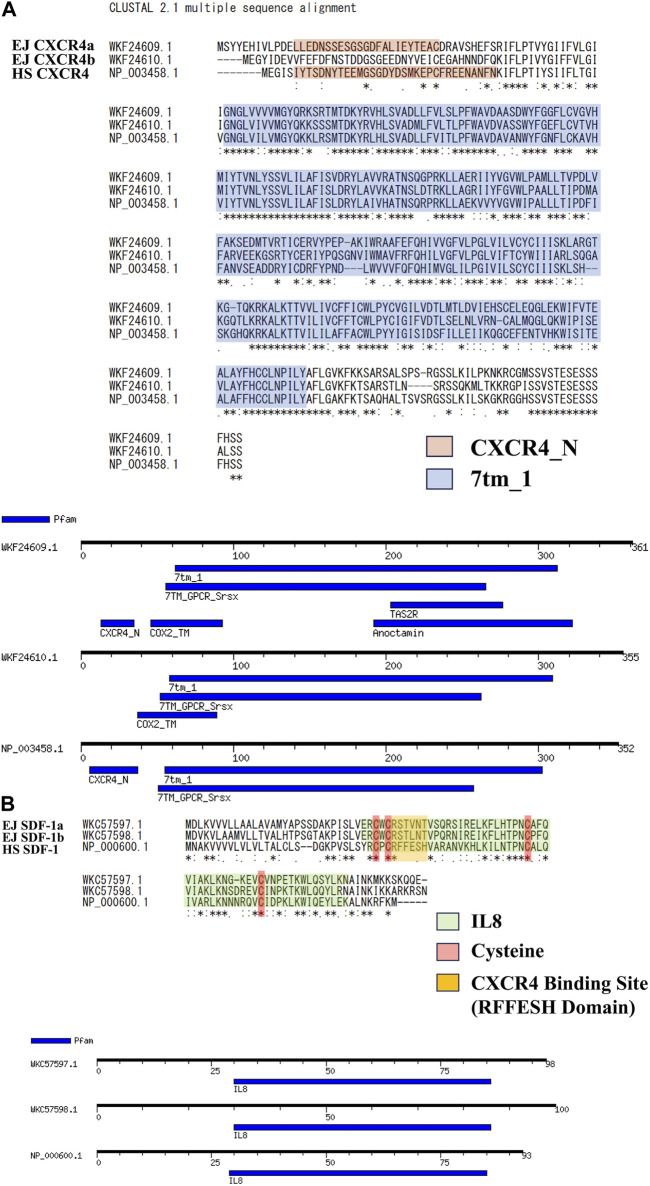
Amino acid sequence analysis for SDF-1/CXCR4 signaling of Japanese anchovy. **(A)** Alignment of CXCR4 amino acid sequence and motif analysis. Sequences of CXCR4_N Motif are shown in orange color, and 7tm_1 Motif are shown in blue color. **(B)** Alignment of SDF-1 amino acid sequence and motif analysis. IL8 Motifs are shown in green, four cysteines and RFFESH domains shown in red and yellow, respectively.

Specific genome duplications have been found in many bony fishes (Wittbrodt et al., 1998; Hoegg et al., 2004; Glasauer and Neuhauss, 2014) including Japanese anchovy. We have isolated two SDF-1 and CXCR4 paralogs (EJ SDF-1a/b, EJ CXCR4a/b) and deposited in NCBI. Based on this, we compared the amino acid sequence of human SDF-1/CXCR4 (HS SDF-1/CXCR4) (Figure 1) and found similarity scores of more than 60 for CXCR4 (Figure 1A) and 40 for SDF-1 (Figure 1B) paralogs. Motif searches suggested that CXCR4 Chemokine receptor N terminal domain (PF12109), required for SDF-1 binding is preserved in EJ CXCR4a, while a deletion was suspected in CXCR4b (Figure 1A), that is same as in zebrafish CXCR4 paralogs (DR CXCR4a/b) (Supplementary Figure S2A). The 7 transmembrane receptors (rhodopsin family, PF00001), characteristic of G-protein coupled receptors (Jacoby et al., 2006), were conserved in both EJ CXCR4a and EJ CXCR4b. Additionally, EJ CXCR4a showed to have more domains similar to other receptors such as TAS2R (bitter taste receptors), Anoctamin (chloride channel), and Cox2 (Cyclooxygenase2). Furthermore, large differences were observed in the amino acid sequence of SDF-1 between Japanese anchovy and humans. The putative RFFESH domain, which is essential for CXCR4 binding, exhibited high similarity between EJ SDF-1a and b, and consistent with zebrafish SDF-1a/b (DR SDF-1a/b) (Supplementary Figure S3), but differed from HS SDF-1. On the other hand, it is noteworthy that the presence of four functional cysteines and the IL8 motif (PF00048, Figure 1B), crucial for CXCR4 binding, remained highly conserved.

### 3.2 Protein analysis and 3D modelling

**FIGURE 2:**
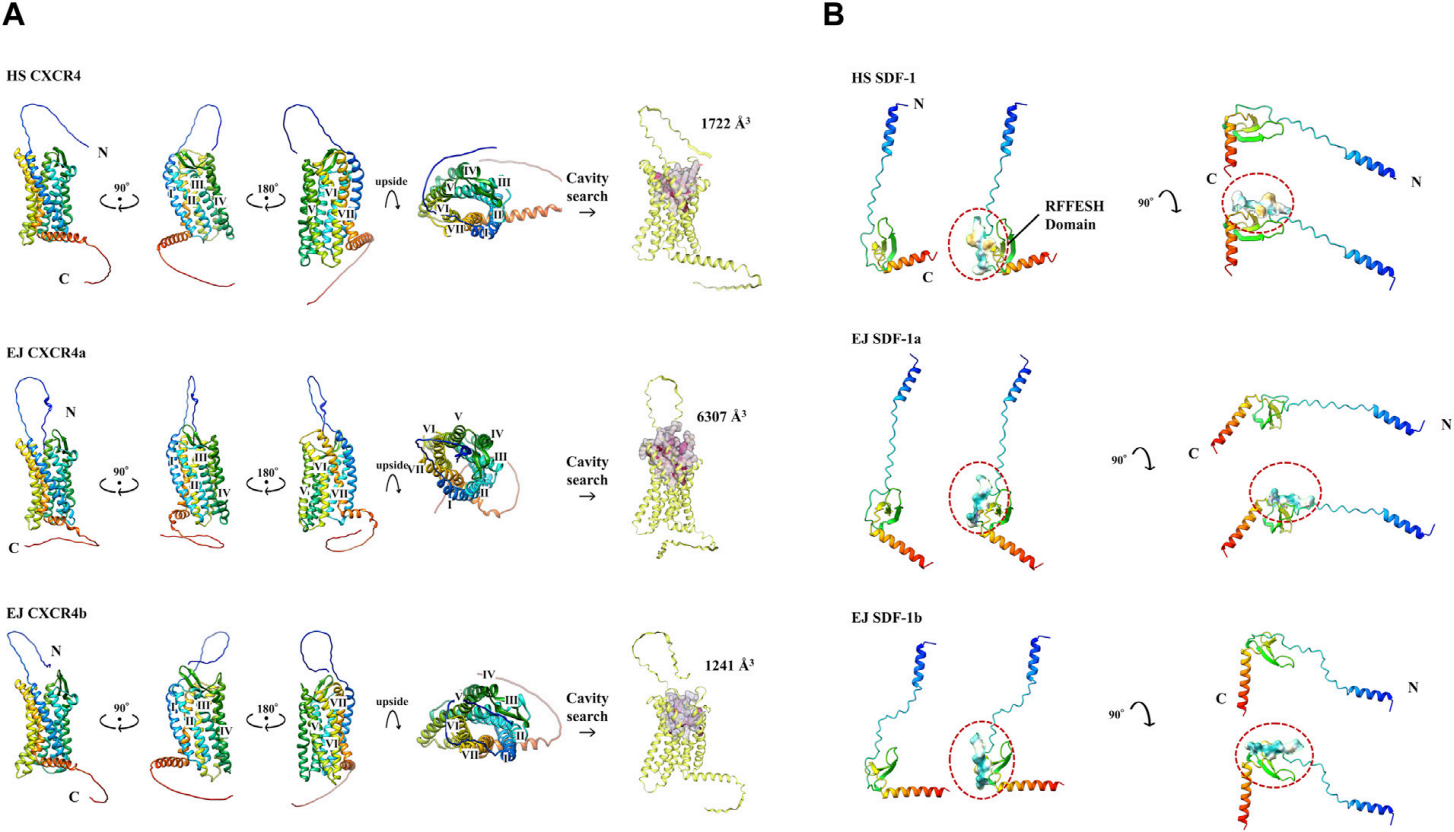
Various protein 3D models and cavity analysis. Protein models are shown in cartoon and rainbow color (N to C, Blue to Red). **(A)** 3D model of CXCR4 protein. N-terminal and C-terminals are marked with N and C, respectively. 7 transmembrane α-helices are indicated from I to VII, respectively. The cavity size (Å^3^) obtained at CB Dock2 of each CXCR4 is shown. **(B)** 3D model of SDF-1 protein. The RFFESH domain, which is required for CXCR4 binding, are additionally shown with the surface, and circled in red.

We used Colab Fold (Mirdita et al., 2022) to construct 3D models of each protein (Figure 2). Result of EJ CXCR4a/b, HS CXCR4, and DR CXCR4a/b protein conformations (Figure 2A, Supplementary Figure S2A) suggest high similarity across these three species, with a typical 7-transmembrane α-helix and a CXCR4 N-terminal loop toward the outer cell membrane. Cavity searches for EJ CXCR4a/b and HS SDF-1 revealed that the size of the presumably functional cavities differed significantly, with EJ CXCR4a (6,307 Å^3^) being nearly five times larger than EJ CXCR4b (1,241 Å^3^) and HS CXCR4 (1722 Å^3^). Zebrafish depicted slightly different picutre, with both DR CXCR4a/b having larger cavities (Supplementary Figure S2A). All the examined SDF-1 protein 3D structures exhibited a distinctive binding site (RFFESH Domain) at the center of the 3D model (Figure 2B, Supplementary Figure S2B), signifying domain conservation. However, ligand (EJ SDF-1a/b) - receptor (EJ CXCR4a/b) binding models (Supplementary Figure S4) were largely different from human counterpart (Phanourios and Christodoulos et al., 2014) and require further investigation.

**FIGURE 3:**
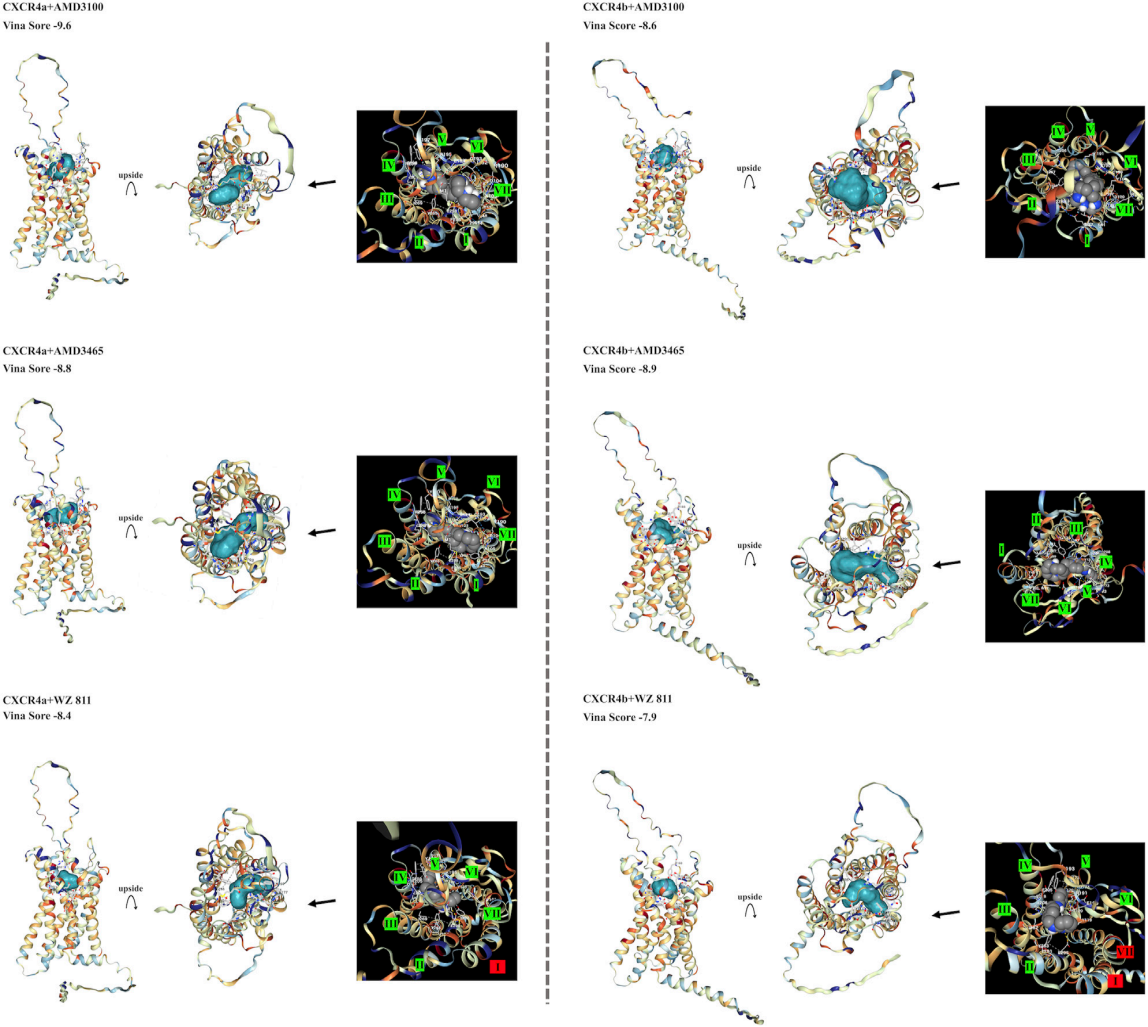
EJ CXCR4 and antagonist binding model of each combination. Vina score, and detail pictures of the binding sites are shown. CXCR4 are shown in cartoons colored with hydrophilicity, and antagonists are shown in the surface colored with blue. In the binding site details, helix numbers with cross-links are shown in green, and non-contacting helices are shown in red.

To select antagonists, a simple simulation binding model was created (Figure 3) using CB Dock. For LY2510429 (MW = 1,189.45 Dalton), CB Dock could not be performed because of its large structure, while AMD3100, AMD3465, and WZ811 possessed a size of 794.48, 896.08 and 290.36 Da, respectively. Results of auto blind docking for three remaining antagonists show relatively higher binding capacities. For EJ CXCR4a, the lowest Vina Score (lower energy required for binding, an indication of easier binding) was AMD3100 (−9.6), followed by AMD3465 (−8.8) and WZ811 (−8.4). On the other hand, EJ CXCR4b demonstrated the strongest binding affinity for AMD3465 (Vina Score −8.9) followed by AMD3100 (−8.6) and WZ811 (−7.9). The location of the binding of AMD3100 and AMD3465, which are close in molecular weight size, shows that they bind well to the 7 TM motif, and could be confirmed with each primordial binding to each TM, regardless of CXCR4a or CXCR4b. On the other hand, WZ811 showed the highest Vina Score, and seemed to have space in the binding site with the 7 TM motif, perhaps due to its smaller size. In fact, no binding was observed (Figure 3; red background) for the 1 TM in CXCR4a and for the 1 and 7 TM in CXCR4b, suggesting that the effect may be weak or easy to remove.

### 3.3 Microinjection based evaluation of antagonist

**FIGURE 4:**
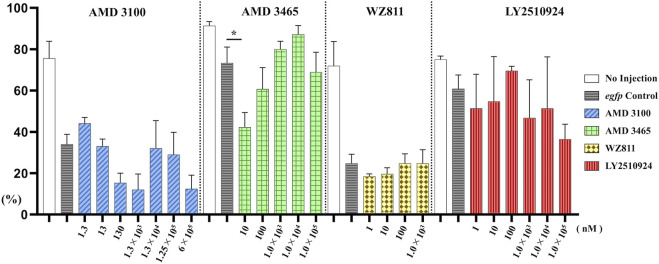
Hatchability (%) at 48 hpf in microinjection experiment. *n* = 31∼105, One-way ANOVA, Tukey test, *: *p* < 0.05.

**FIGURE 5:**
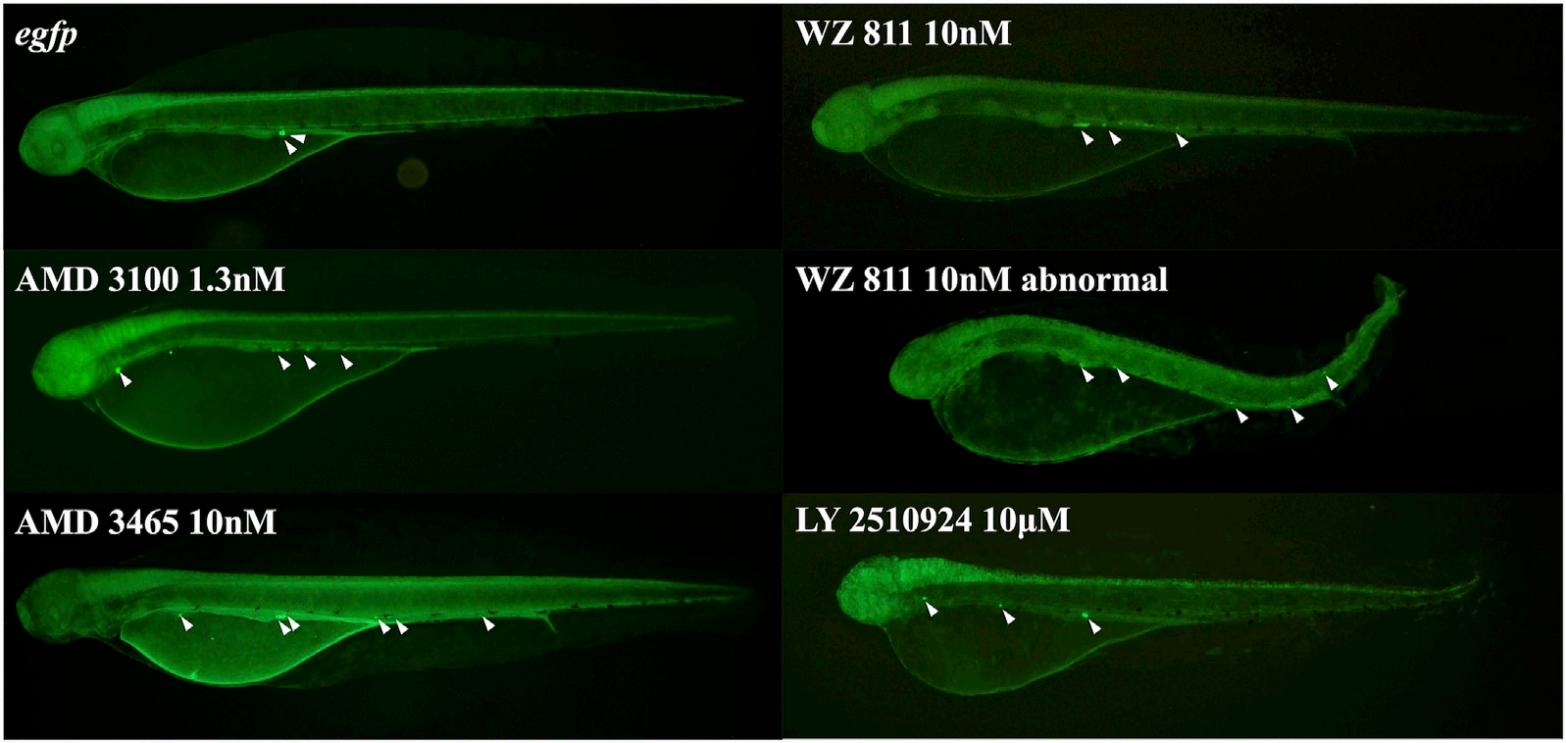
Fluorescence observation at 48 hpf of antagonist microinjection experiment. White arrows indicate the location of PGC, that specifically visualized by *egfp-nanos3* 3′UTR mRNA. PGCs are dispersed throughout the body in the microinjected group.

**FIGURE 6:**
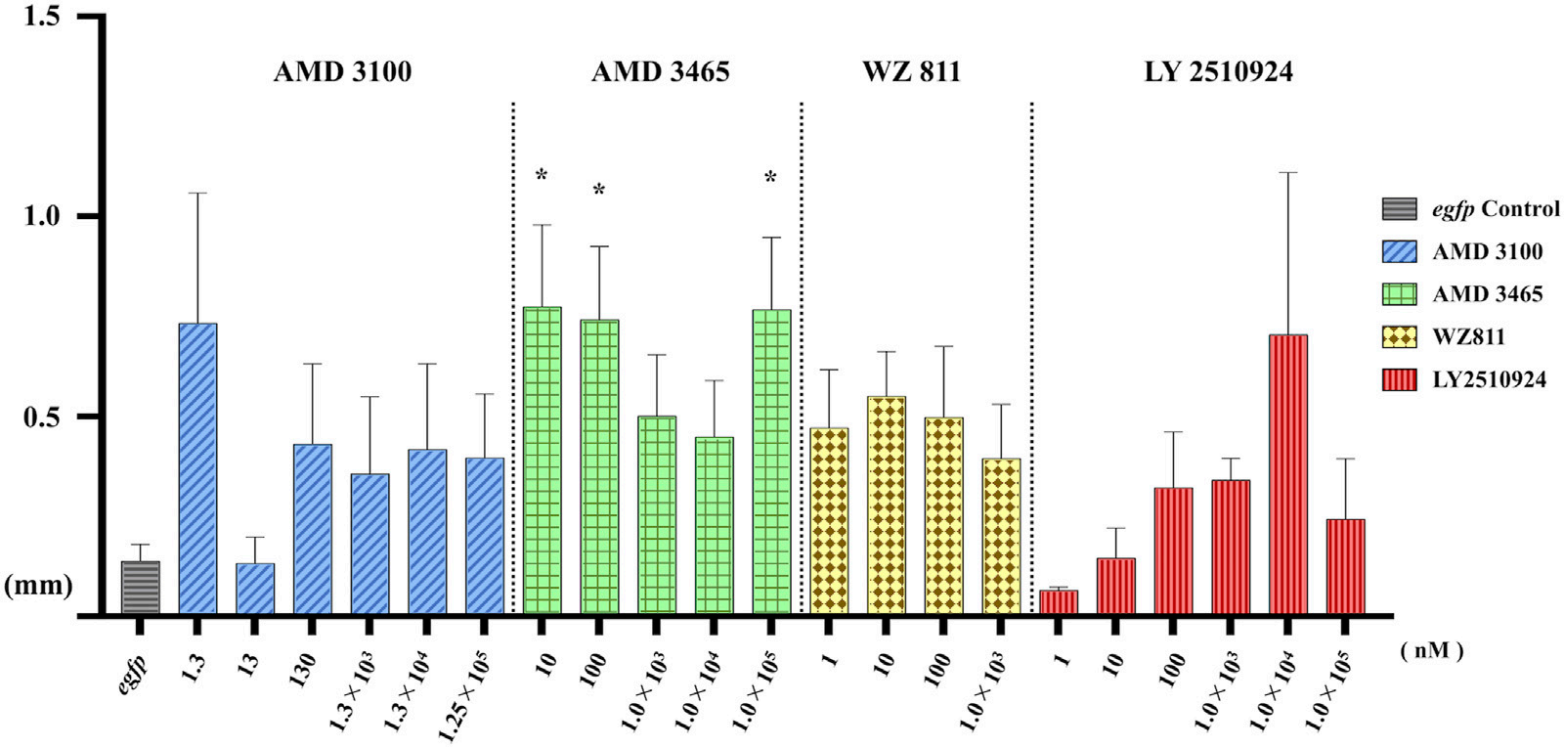
Distance between the Farthest PGCs (DFP, mm) analysis at 48 hpf of antagonist microinjection experiment. A significant increase compare with control was found only for AMD 3465. *n* = 31∼105, One-way ANOVA, Tukey test, *: *p* < 0.05.

To directly introduce the antagonist into the eggs and determine its effect on PGC, we microinjected the antagonist into fertilized eggs, measured the hatchability at 48 hpf (Figure 4), and compared with the solvent injected (*egfp* control) and non-injected controls. The results showed that hatchability decreased in both injected groups compared to their respective non-injected controls. Lowest hatchability was observed in WZ811 treatment groups and DMSO (solvent control of WZ811) treated individuals. However, no significant difference was recorded in antagonist-treated groups compared to their *egfp* counterpart, across different concentration (except for AMD3465, 10 nM). Apart from WZ811 treated and DMSO treated fish, no external malformation was observed. These results suggest that none of the antagonists are biotoxic, and the reduced hatchability and malformations observed in the WZ811 group are due to solvent (DMSO) effects. Furthermore, fluorescence microscopy depicted that the PGCs were clustered in the putative gonadal ridge of *egfp* group larvae while varying degree of PGC dispersion (characteristics of PGC mismigration) were observed in antagonist-treated individuals (Figure 5). Significantly higher mismigration, i.e., DFP (Figure 6), was observed in AMD3465 treated fish at 10, 100 and 1 × 10^5^ nM concentration. This result corresponds with our *in silico* low Vina score prediction data. Further, as predicted in our *in silico* analysis, significantly different DFP was not observed WZ811 treated fish. Contrastingly, despite low vina score prediction, relative PGC mismigration remained unaffected in AMD3100 treated fish across various doses. Additionally, about LY2510924 treatment groups, a dose dependent escalation in DFP, except at 1 × 10^5^nM, was observed.

### 3.4 Evaluation of antagonist through immersion

**FIGURE 7:**
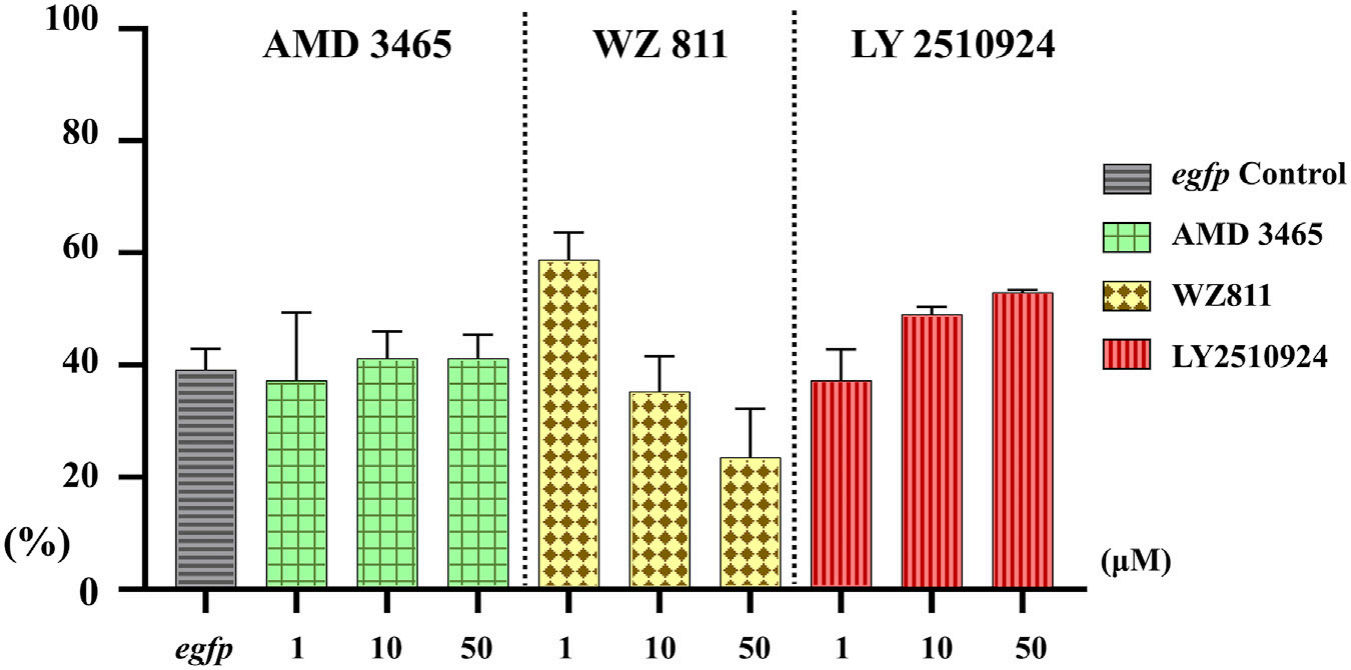
Hatchability (%) at 48 hpf in antagonist immersion experiment. No significant reduction was observed. *n* = 17, One-way ANOVA, Tukey test.

**FIGURE 8:**
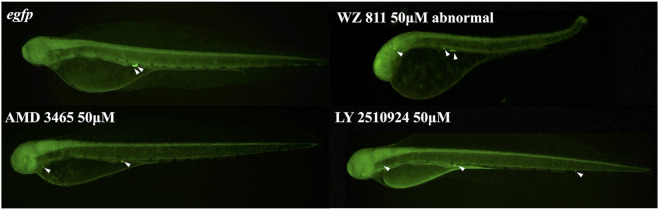
Fluorescence observation at 48hpf of antagonist immersion experiment. White arrows indicate the location of PGC. PGCs are dispersed throughout the body in the antagonist immersion groups.

**FIGURE 9:**
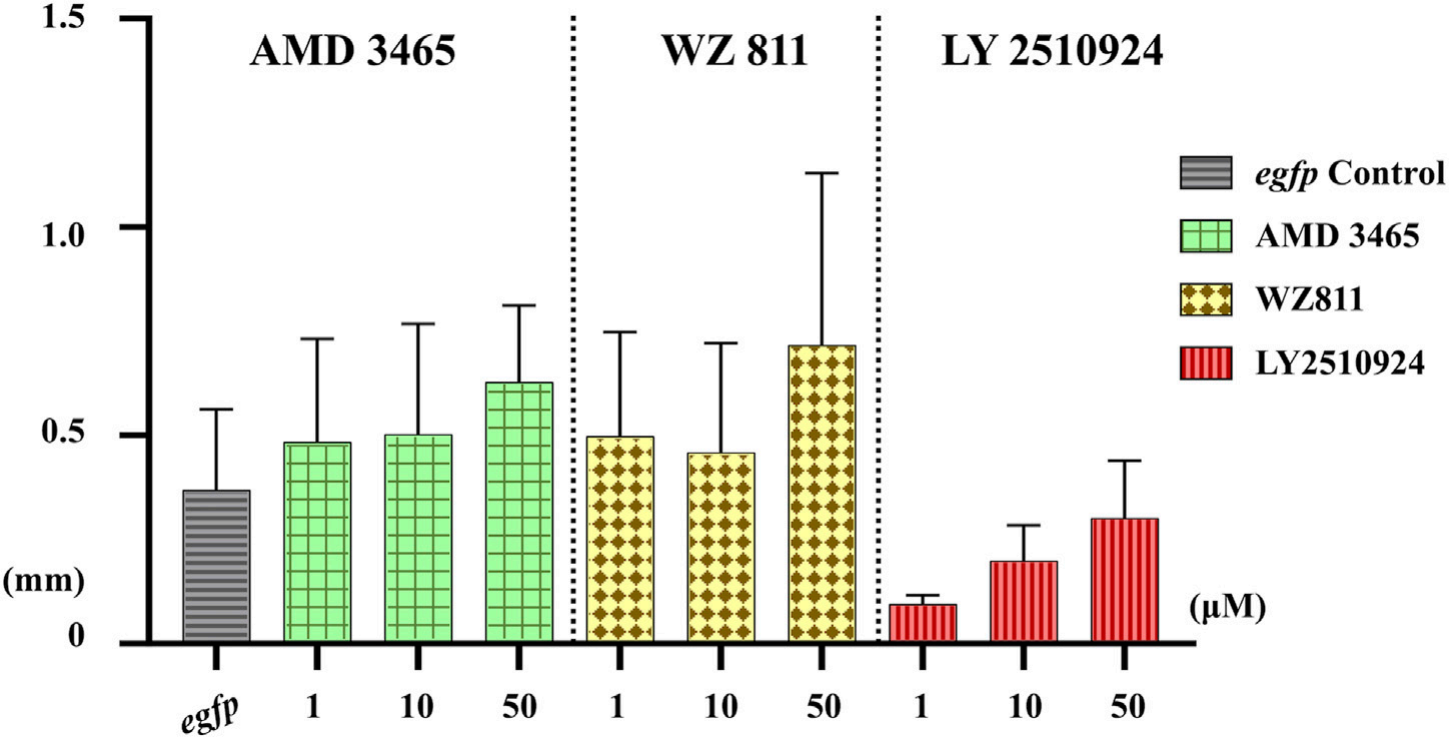
Distance between the Farthest PGCs (DFP, mm) analysis at 48 hpf of antagonist immersion experiment. No significant difference was observed among the groups. *n* = 6, One-way ANOVA, Tukey test.

Microinjection method ensures direct delivery into the eggs, but significantly damages the egg membranes, resulting in poor survival rates. It also demands a great deal of technician skill and time, greatly limiting the number of eggs that can be processed. Therefore, a simpler and more practical method is needed for mass scale treatment in actual aquaculture. We employed immersion treatment using AMD3465, WZ811 and LY2510924 only. Similar to direct delivery microinjection experiment, no significant decrease in hatchability (Figure 7) and in any of the antagonist groups was observed. However, an inverse dose dependent trend was observed in WZ811 treatment group due to the different amounts of solvents used. As seen with microinjection, at 48 hpf PGCs were largely dispersed (Figure 8) in antagonist treated individuals with highest DFP (Figure 9) average of 0.71 mm followed by 0.62 mm and 0.50 mm in 50 µM-WZ811, 50 μM-AMD3465 and 10 μM-AMD3465 treatment groups, respectively. On the other hand, the LY2510924 group showed a reduced DFP. Overall, unlike microinjection, DFP analysis showed no significant differences among all immersion treatment groups.

## 4 Discussion

In this study, we focused on screening and validating better CXCR4 antagonist to control the SDF-1/CXCR4 associated PGC migration in Japanese anchovy. We have created a simplified 3D model of SDF-1 and CXCR4, and clarify the *in vivo* effects of pharmaceutical grade CXCR4 antagonists on PGC migration.

Until recently, obtaining an accurate 3D model of protein required heavy work, such as creating crystals and obtaining their X-ray diffraction, which is extremely difficult to apply to non-model organisms since there are still many unresolved proteins. On the other hand, the accuracy of Alpha Fold2, which makes use of recent computational methods and deep learning to generate protein structure models (Senior et al., 2020; Jumper et al., 2021), has improved dramatically. Furthermore, with the advent of Colab Fold, which can be used quickly and easily on an online server, it is now possible to easily obtain 3D models of non-model organisms, even if one is not a protein structure scientist. In this study, we used these tools to obtain simplified 3D models of EJ SDF-1a/b and EJ CXCR4a/b, then compare with the human and zebrafish models.

For EJ SDF-1a/b, close 3D models were obtained, even though the amino acid sequence was less conserved with HS SDF-1. With partial comparisons, the yielded models exhibited a high degree of approximation after the CXCR4 binding site (RFFESH motif, Crump et al. (1997), Smith et al. (2014)), suggesting that the SDF-1 protein is relatively conserved in all three species. However, both models unlike previously published crystal structure models (PDB ID: 3GV3, Murphy et al. (2010), Pawig et al. (2015)), N-terminus is not attracted to the binding site and is wide open. For EJ CXCR4a/b, with a well-conserved amino acid sequence, 3D models were obtained with a high degree of approximation, suggesting conserved CXCR4 activity across species. Interestingly, our *in silico* data shows that EJ CXCR4a possesses conserved CXCR4 N-terminal motif but shows mutations in the N-terminal α-helix structure (Supplementary Figure S5). EJ CXCR4a and DR CXCR4a/b had an extremely large cavity, about 2∼5 times larger than that of EJ CXCR4b and HS CXCR4. In contrast, EJ CXCR4b has a high 3D structural similarity of the N-terminus despite the loss of the CXCR4 N-terminal motif, and a comparable cavity size to that of HS CXCR4. These suggest that SDF-1/CXCR4 signaling is relatively conserved between Japanese anchovy, zebrafish, and humans, but the EJ SDF-1 and EJ CXCR4 paralogs have distinct actions.

Supporting this, we earlier reported a similar expression profile of *cxcr4a* and *cxcr4b* during early embryogenesis, with *sdf-1a* expression preceding *sdf-1b*. Additionally, germ cell-specific overexpression of *sdf-1a* resulted in decreased expression of *cxcr4a* and increased expression *cxcr4b* in Japanese anchovy (Yahiro et al., submitted elsewhere). Furthermore, the results in zebrafish (*Danio rerio*) and medaka (*Orizias latipes*) showed that SDF-1a and CXCR4b are essential for proper positioning and bilateral lining of PGC (Raz, 2003; Sasado et al., 2008), and the main ligand of CXCR4b is SDF-1a (Doitsidou et al., 2002; Dambly-Chaudière et al., 2007). These suggest that SDF-1/CXCR4 signaling in Japanese anchovy is similar with zebrafish and medaka. It might be reasonable to consider that after the fish-specific genome duplication event, EJ CXCR4b retained its roles related to PGC migration through interaction with EJ SDF-1a, similar to other vertebrates. Conversely, there is some possibility that EJ CXCR4a changes in form and acquired additional functions. To support this hypothesis, we tried to generate SDF-1/CXCR4 protein binding 3D model (Supplementary Material S1) by Alpha Fold2 Multimer. A recent study used Alpha Fold2 Multimer to predict peptide-protein binding models and obtained high accuracy (Evans et al., 2021; Johansson-Åkhe and Wallner, 2022). Since EJ SDF-1a/b are small proteins (<100 aa), we applied a similar method but failed to obtain a model that binds to the N-terminus of CXCR4, despite SDF-1 conserved RFFESH motif. Further in-depth analysis will be necessary in the future.

On the other hand, due to the significant similarity between human and Japanese anchovy CXCR4 3D models, we predicted the CXCR4-antagonist (receptor-ligand) interaction for well-known pharmaceutical-grade human CXCR4 antagonists. Three out of four ligands showed excellent modeling but had different Vina scores among two homologs, probably due to different cavity conformation. For instance, EJ CXCR4a had the lowest Vina score for AMD3100, while EJ CXCR4b exhibited the lowest Vina score with AMD3465. The Vina scores alone suggest that EJ CXCR4a and EJ CXCR4b respectively prefers AMD3100 and AMD3465. Moreover, our structural analysis shows that, EJ CXCR4b has a comparable cavity (in terms of size) with HS CXCR4. So, it is highly likely that, CXCR4b might play a major role in PGC migration in Japanese anchovy and germ cell mismigration will be higher in AMD3465. Our data shows that, in agreement with above hypothesis, microinjection of AMD3465 resulted in the significantly largest DFP, followed by AMD3100. This result also enhances the possibility that EJ CXCR4b inhibition might have an upper edge in PGC mismigration, largely highlighting the reliability of the computational analysis presented in this study.

Effective drug delivery is essential for mass production, but we obtained better outcome with direct delivery of CXCR4 antagonists into the eggs. Immersion treatment was relatively less effective. For instance, highest DFP was 0.77 mm after injection of 10 nM AMD3465, but even with a 5,000 times higher dose through immersion, it could only deflect the PGC by 0.62 mm. Such differences might be attributed to variations in the composition and permeability of the egg envelope proteins. A dose-responsive increasing trend in antagonist responsiveness was observed in the immersion treatment, suggesting that a higher concentration might be necessary to achieve a significant result. Considering large scale operation and environment preservation alternate strategies like altered water temperature, mild electric pulse might be explored in future.

In summary, we have combined *in silico* and *in vivo* analysis to characterize SDF-1/CXCR4 signaling in Japanese anchovy and identify more suitable antagonist from readily available pharmaceuticals. We found that AMD3465, probably via CXCR4b, alters SDF-1/CXCR4 chemotaxis and disrupts PGC migration in Japanese anchovy. Expectedly, this investigation will be a valuable step towards the development of simple, versatile, and low-cost gonad sterilization technology in actual aquaculture condition.

## Data Availability

The datasets presented in this study can be found in online repositories. The names of the repository/repositories and accession number(s) can be found below: https://www.ncbi.nlm.nih.gov/, WKC57597.1 https://www.ncbi.nlm.nih.gov/, WKC57598.1 https://www.ncbi.nlm.nih.gov/, WKF24609.1 https://www.ncbi.nlm.nih.gov/, WKF24610.1.
