# Supplementary figures and images for: Modeling the SDF-1/CXCR4 protein using advanced artificial intelligence and antagonist screening for Japanese anchovy
Source: Front Physiol. 2024 Feb 2;15:1349119. doi: 10.3389/fphys.2024.1349119 (PMC10869568; doi:10.3389/fphys.2024.1349119)

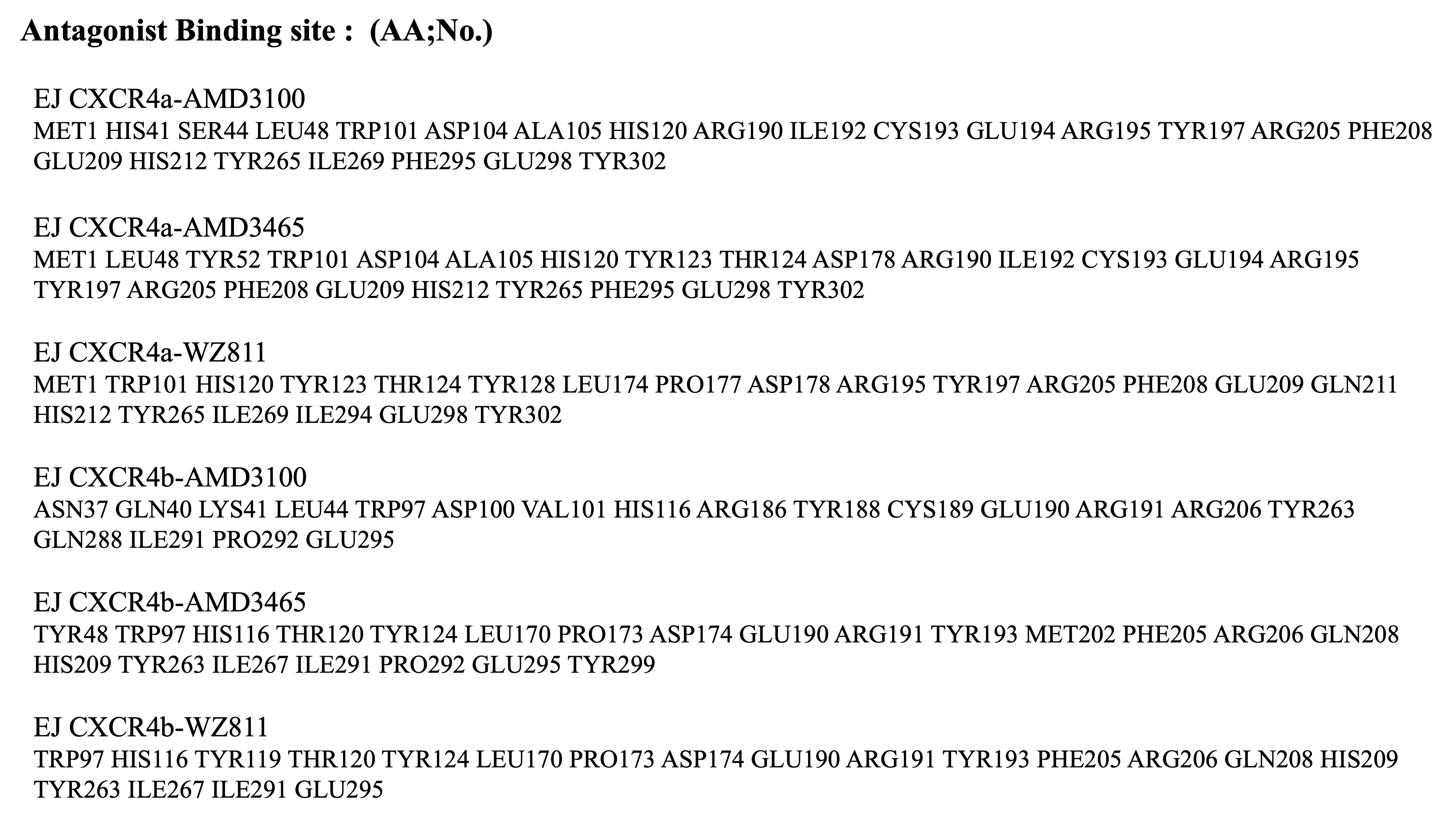

Supplement: Supplementary file 1 [file DataSheet1.zip › Supplement figure 1.tiff]

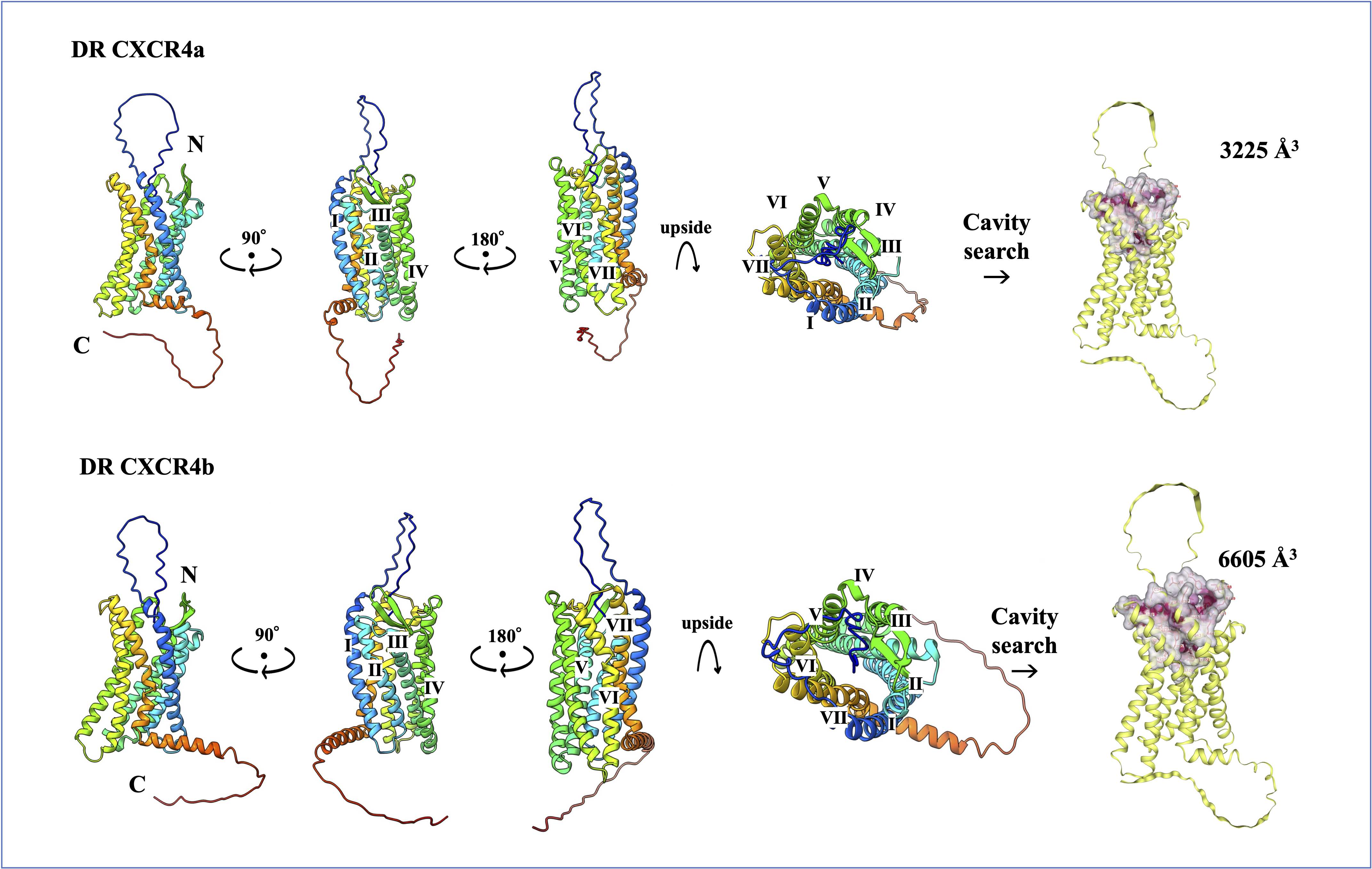

Supplement: Supplementary file 1 [file DataSheet1.zip › Supplement figure 2A.tiff]

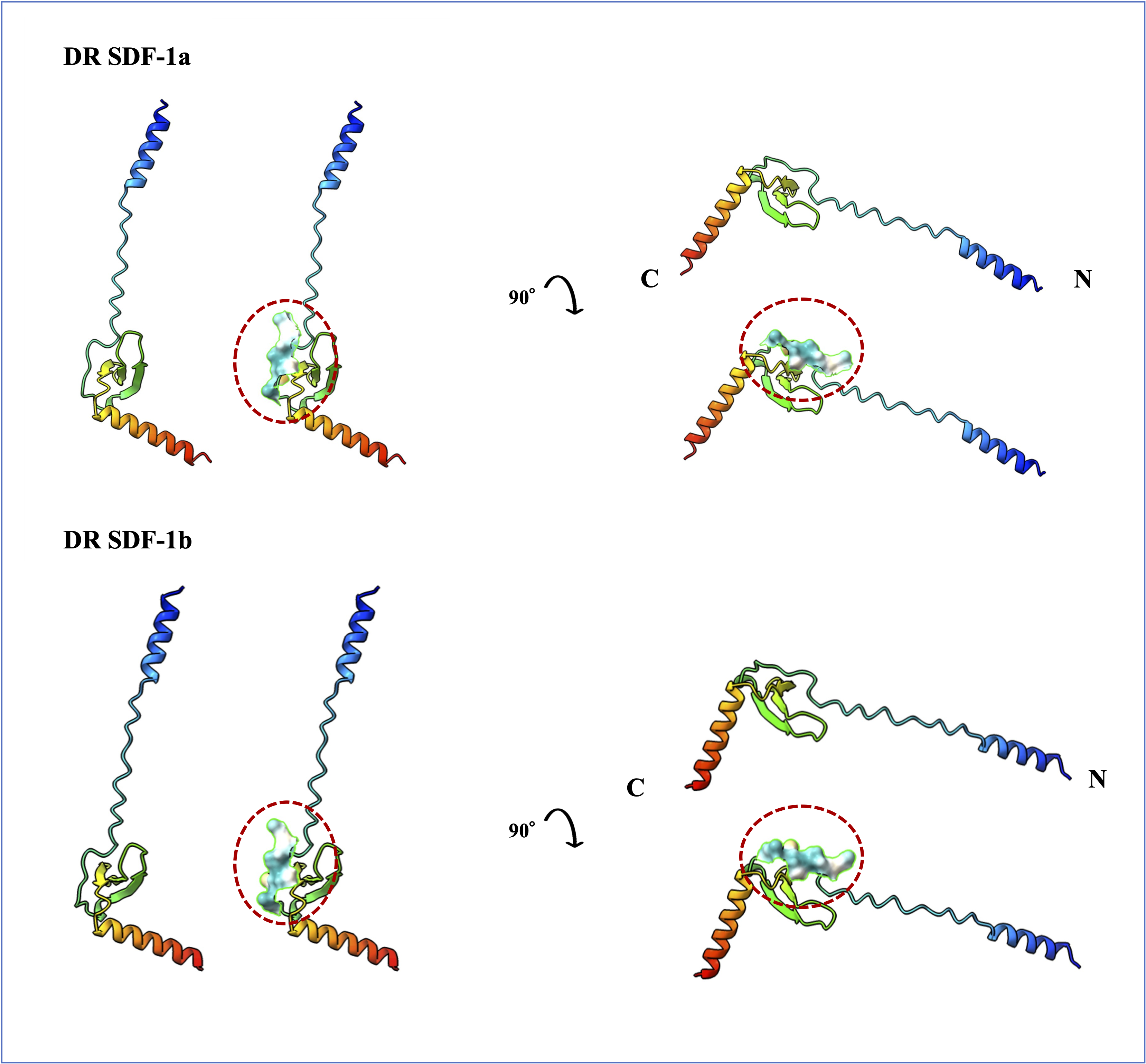

Supplement: Supplementary file 1 [file DataSheet1.zip › Supplement figure 2B.tiff]

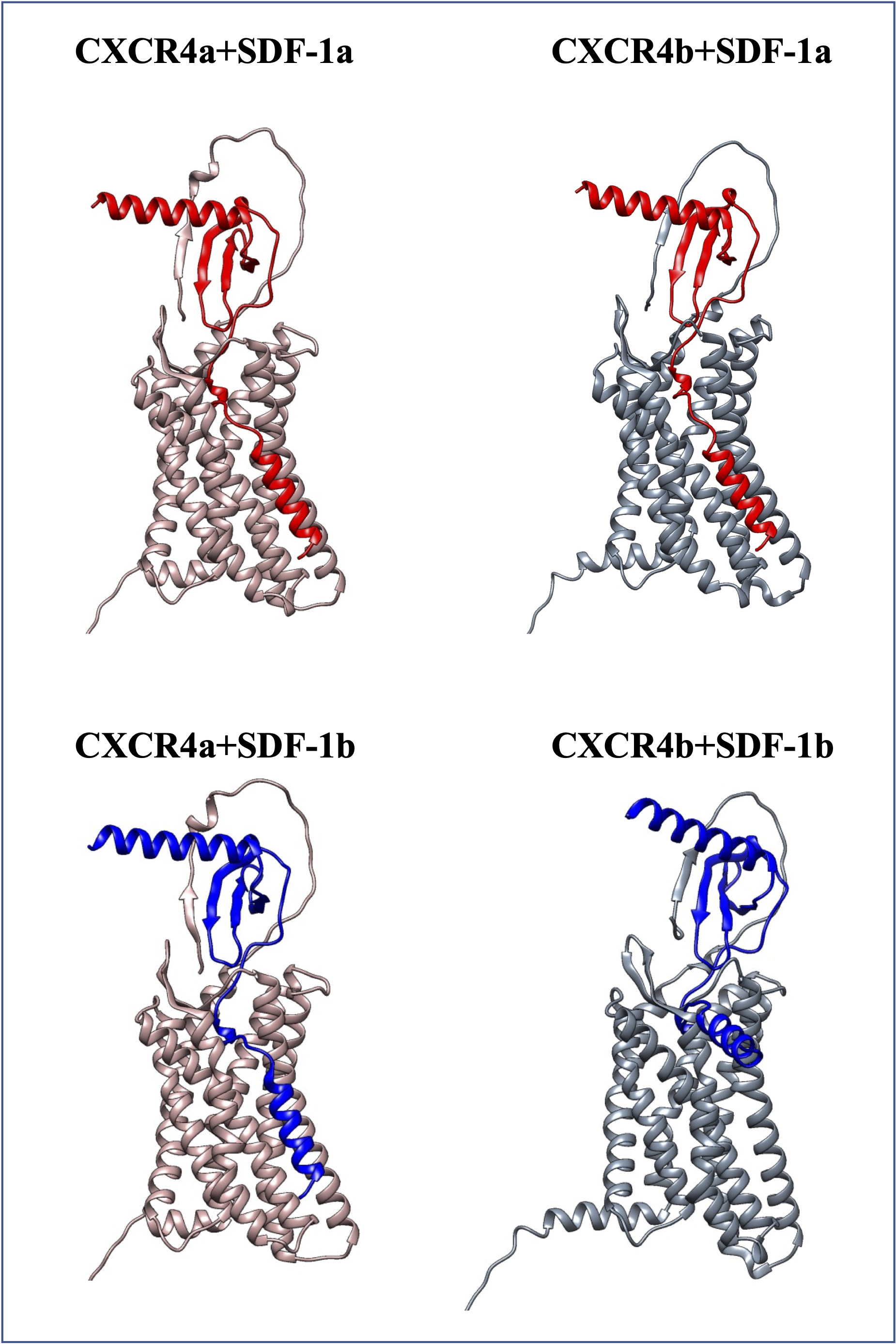

Supplement: Supplementary file 1 [file DataSheet1.zip › Supplement figure 4.tiff]

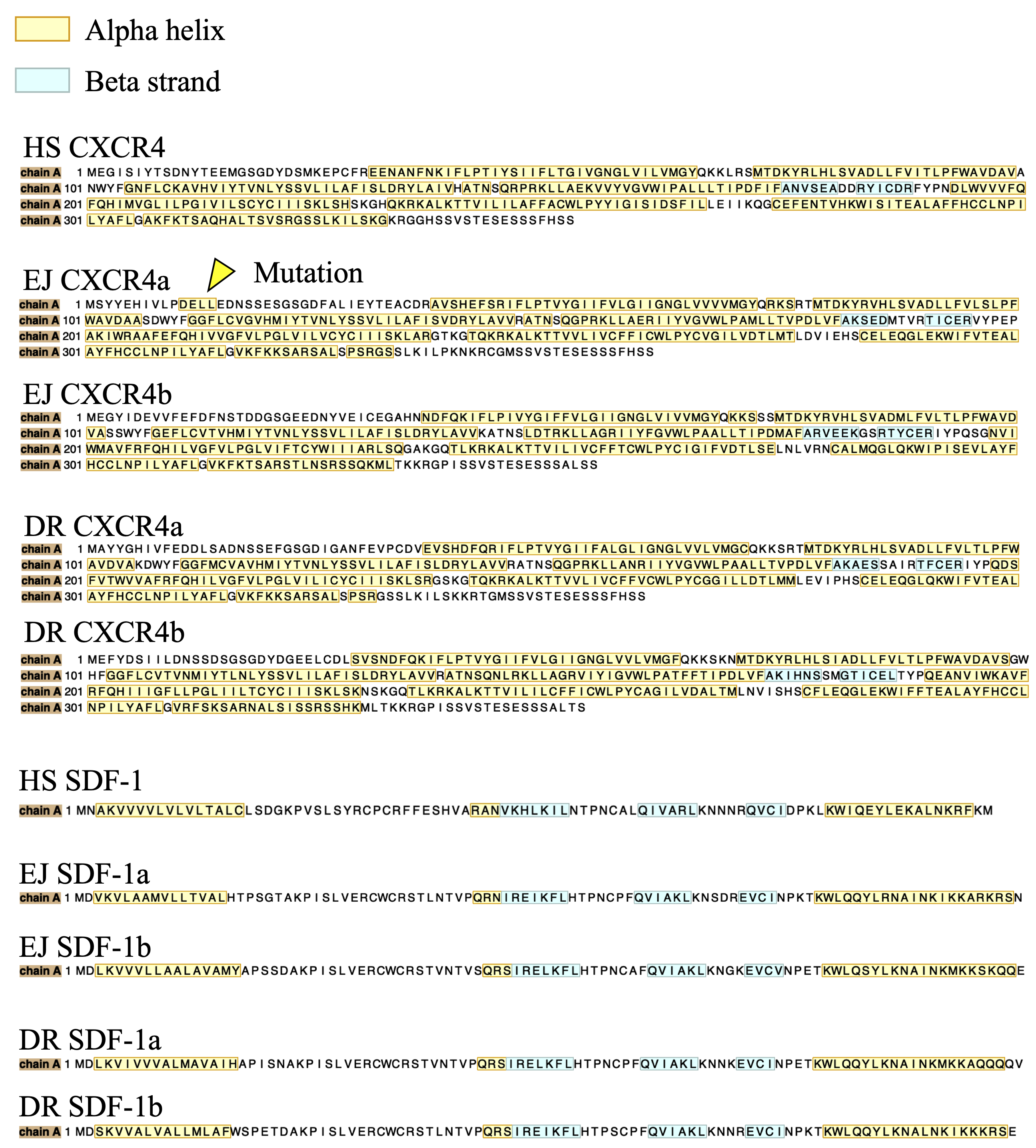

Supplement: Supplementary file 1 [file DataSheet1.zip › Supplement figure 5.tiff]
